# Bevacizumab based chemotherapy in first line treatment of HER2 negative metastatic breast cancer: results of a Moroccan observational institutional study

**DOI:** 10.1186/1756-0500-5-162

**Published:** 2012-03-22

**Authors:** Lamiaa Boulaamane, Saber Boutayeb, Hassan Errihani

**Affiliations:** 1 Department of medical oncology, National institute of oncology, 10100 Rabat, Morocco; 2 Department of Medical Oncology, National Institute of Oncology, Sidi Mohamed Ben Abdellah Hospital, Rabat, Morocco

**Keywords:** Bevacizumab, Metastatic breast cancer, Taxanes, Meta-analysis, Salvage treatment

## Abstract

**Background:**

In metastatic breast cancer (MBC) patients, randomised controlled trials evaluated Bevacizumab as first-line treatment showed improvements in tumour response rate and progression-free survival (PFS) when added to chemotherapy. In Morocco, we conducted an observational study to investigate clinical features, treatment and prognosis associated with Bevacizumab based chemotherapy in first line treatment of HER2 negative MBC.

**Findings:**

Nineteen women were included in this study. All these women were diagnosed as having HER2 negative MBC at the National Institute of Oncology in Rabat, Morocco, between January 2009 and December 2010. The median age of patients was 48.1 years. Four patients (21%) had metastatic disease at diagnosis and 15 patients (79%) had received treatment for first metastatic relapse. Bone, liver and lung were the most frequent metastasis sites. Patients were followed up until April 2011. Most patients had objective response; 15.8% of complete response, 47.3% of partial response and 21.1% of stabilisation. Median PFS was estimated at 11.5 months. Sub-groups analysis showed a statistically significant difference (Log-rank test: p = 0.01); PFS for patients receiving Bevacizumab - weekly Paclitaxel was estimated at 18.1 months, and at 9.1 months for patients receiving the combination Bevacizumab - Docetaxel. This benefit in PFS was associated with an acceptable safety profile.

**Conclusion:**

As demonstrated in this study, Bevacizumab based chemotherapy in first line treatment of HER2 negative MBC in Morocco and particularly in combination with Taxanes extends PFS, as confirmed in a recent meta-analysis of 3 randomised controlled phase III studies.

## Background

Metastatic breast cancer (MBC) is rarely cured, and median survival after the development of metastatic disease is only 2 to 3 years [1]. Although many chemotherapeutic agents are available for the treatment of MBC, the median survival duration has not improved until very recently.

Numerous studies have demonstrated that angiogenesis and in particular VEGF (vascular epidermal growth factor) over-expression play an essential role in the growth, progression and metastatic potential of breast cancer [2-5]. Thereafter, VEGF became a fundamental target of anti-angiogenic therapy.

Bevacizumab is a humanised recombinant monoclonal antibody that specifically blocks the binding of VEGF to high-affinity receptors [6]. In MBC patients, randomised controlled trials evaluated Bevacizumab as first-line treatment showed improvements in tumour response rate and progression-free survival (PFS) when added to chemotherapy [7-9]. However, none of the trials showed significant survival benefit. More recently, a meta-analysis of three randomised, controlled, phase III trials confirmed that the addition of Bevacizumab to chemotherapy regimens provides substantial benefit for women with MBC in terms of PFS and objective response (OR), but not in overall survival (OS)[10].

The aim of our work is to report histological and therapeutic characteristics, to describe OR and safety profile, and mainly to analyse PFS of patients treated with Bevacizumab-based chemotherapy in first line treatment of HER2 negative MBC, especially in Moroccan population.

## Patients and methods

### Clinical data

This is an observational institutional study. We searched the patient records at the Department of Clinical Oncology, in the national institute of oncology of Rabat, for the period from January 2009 to December 2010 for all tumours coded as HER2 (human epidermal growth factor receptor) negative MBC. The study respected the ethical rules for medical research involving human subjects as stipulated by the WMA Declaration of Helsinki. Our local hospital committee has approved this study and patients gave their consent. The files of 19 HER2 negative MBC patients treated at our institution were thoroughly analysed.

All patients had an histologically proven diagnosis of breast cancer. HER2/neu receptor status was evaluated using IHC (Immunohistochemistry) or FISH (Fluorescence in situ hybridisation). HER2/neu oncoprotein expression negativity was assessed using the Hercep Test, scored 0 (absent) or 1+ (weak), and the negativity of the HER2/neu gene amplification was confirmed by FISH if score 2+ (moderate) in Hercep Test.

This study had excluded HER2 positive MBC, and all patients receiving already first line treatment of MBC with or without Bevacizumab.

Among 19 patients treated for HER2 negative MBC, 4 (21%) had metastatic disease at diagnosis and 15 (79%) had received treatment for first metastatic relapse (after a median time of 32.8 months from adjuvant treatment of localized disease).

### Follow up

Patients were followed up until April 2011. All patients who were not reviewed in the last consultation were contacted again by telephone.

Assessment of response was performed using RECIST (Response evaluation criteria in solid tumours) criteria. A complete response (CR) was defined as the complete disappearance of all evidence of disease. A partial response (PR) was defined as a reduction of at least 30% of the sum of the largest diameters of each target lesion, without the appearance of new lesions. Progression was defined as an increase of at least 20% of the sum of the largest diameters of each target lesion, or the appearance of one or more new lesions.

Progression-free survival was calculated from the date of start of treatment until progression or until the date of last visit.

### Statistical analysis

Data was analysed using Win-hospital; an electronic CRF (case report form). Descriptive statistics with 95% confidence interval (CI) were calculated according to standard procedure. Survival curves were constructed using the Kaplan-Meier method. The log-rank test was used to compare survival curves. The test was conducted at a 5% significance level.

## Findings

The median age of patients was 48.1 years. Infiltrating ductal carcinoma was the predominant histological subtype, and the majority of patients had SBR (Scarff, Bloom and Richardson) grade II of tumours (47.3%) and positive hormone receptors (63.1%) (Table 1).

**Table 1:** Clinico-pathological characteristics of patients

Median age	48.1 years
**Histological type**	Infiltrating ductal carcinoma
**SBR grade**	
I	2 (10.5%)
II	9 (47.3%)
III	5 (26.3%)
Not available	3 (15.7%)
**Hormone receptors**	
Positive HR	12 (63.1%)
Negative HR	6 (31.5%)
Not available	1 (5.2%)

As indicated in Table 2 below, metastasis site was variable; bone (63.1%), liver (47.3%) and lung (47.3%) were the most frequent. However, it should be stressed that percentages in Table 2 do not add up to 100% because of overlapping

**Table 2:** Metastasis site

Metastasis site	Number of patients
Bone	12 (63.1%)
Liver	9 (47.3%)
Lung	9 (47.3%)
Local recurrence	5 (26.3%)
Lymph nods	4 (21%)
Contralateral recurrence	3 (15.7%)
Brain	1 (5.2%)

Chemotherapeutic regimens used were different. Seven patients (36.8%) had received Bevacizumab (10 mg/kg at day 1 and 15) - Paclitaxel (90 mg/m2 at day 1, 8 and 15) every 4 weeks, while 7 other patients (36.8%) were treated with Bevacizumab (15 mg/kg at day 1) - Docetaxel (100 mg/m2 at day 1) every 3 weeks. The combination Bevacizumab (15 mg/kg at day 1) - Paclitaxel (175 mg/m2) every 3 weeks was received by 2 patients (10.5%), and 2 other patients (10.5%) were treated with Bevacizumab (15 mg/kg at day 1) - Capecitabine (1000 mg/m2 from day 1 to 14). Only 1 patient (5.2%) used the protocol Bevacizumab (15 mg/kg at day 1) - Navelbine (25 mg/m2 at day 1 and 8)

The median number of cycles received was 10 cycles (range 1 to 31)

After a median number of 6 courses of Bevacizumab based chemotherapy, most patients had an objective response; 3 patients (15.8%) achieved CR, 9 (47.3%) achieved PR, and 4 patients (21.1%) obtained stabilisation with treatment. However for 3 patients (15.8%), the disease was progressing (Table 3)

**Table 3:** Response assessment to treatment

Response	Number of patients
Complete response	3 (15.8%)
Partial response	9 (47.3%)
Stabilisation	4 (21.1%)
Progression	3 (15.8%)

**Figure 1:**
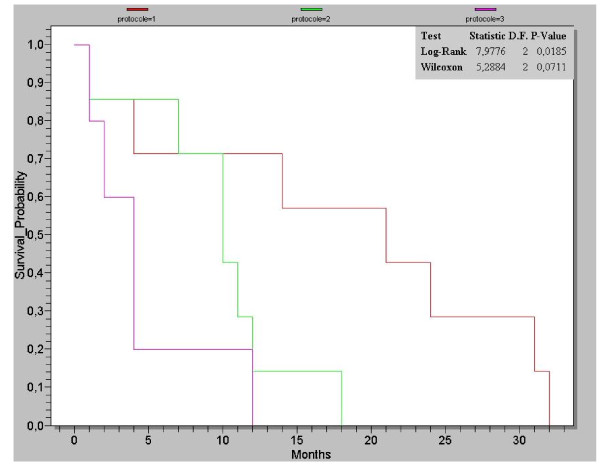
**Kaplan-Meier curve for patient's survival**.

After a median follow-up of 24 months, the median PFS was estimated at 11.5 months. Sub-groups analysis showed a statistically significant difference (Log-rank test: p = 0.01); PFS for patients receiving Bevacizumab - weekly Paclitaxel was estimated at 18.1 months, and at 9.1 months for patients receiving the combination Bevacizumab - Docetaxel (Figure 1)

Bevacizumab based chemotherapy was generally well tolerated (grade 2 hypertension in one patient which was well managed by medical treatment, and slight epistaxis reported by 3 patients). Only one severe toxicity was noted in one patient, represented by grade 3 bloody diarrhoea requiring treatment discontinuation (Table 4)

**Table 4:** Safety profile

Toxicity	Number of patients
Hypertension (grade II)	1(5.2%)
Blood diarrhoea (grade III)	1(5.2%)
Slight epistaxis (grade I)	3(15.8%)

## Discussion

Tumour growth and development are dependent on angiogenesis [11]. Vascular endothelial growth factor (VEGF) is a key pro-angiogenic mediator, overexpressed in many tumours and associated with poor prognosis, making it an attractive target for the development of a biological therapy [12]. Agents that target angiogenesis, a process essential for all types of solid tumour, have the potential to benefit all breast cancer patients, regardless of patient and/or disease characteristics. Inhibition of VEGF signalling has been shown to provide control of tumour growth and metastasis by causing regression of tumour vasculature, normalisation of surviving vasculature and inhibition of further tumour angiogenesis [13,14].

Bevacizumab is a humanised monoclonal antibody that specifically binds VEGF, preventing it from interacting with receptors on vascular endothelial cells and thereby inhibiting its pro-angiogenic effects [15,16]. Bevacizumab is currently approved for the treatment of MBC, metastatic colorectal cancer, non-small-cell lung cancer and renal cell cancer [17,18]. It is also approved in the United States for the treatment of recurrent glioblastoma multiforme [17].

There is preclinical evidence that Taxanes have antiangiogenic, as well as cytotoxic, effects, indicating possible synergies with agents targeting VEGF [19,20]. This has generated much interest in the combination of antiangiogenic biological agents with Taxanes. At present, Bevacizumab is the most advanced anti-VEGF agent in development, with clinical data available from three phase III studies in patients with MBC [7,9,21-23].

E2100 was a phase III trial designed to compare the efficacy of Bevacizumab plus Paclitaxel with Paclitaxel alone in the first-line treatment of locally recurrent (LR) breast cancer or MBC [7,21]. Data from this trial supported regulatory approval of Bevacizumab plus Paclitaxel in Europe and the United States [17,18]. Weekly Paclitaxel (90 mg/m2, weeks 1-3 every 4 weeks) was administered to 722 patients, either alone or with Bevacizumab (10 mg/kg every 2 weeks). Treatment was continued until disease progression or unacceptable toxicity. The combination of Bevacizumab with Paclitaxel significantly increased investigator-assessed PFS compared with Paclitaxel alone [median 11.4 versus 5.8 months; hazard ratio (HR) = 0.42; P < 0.0001] and had a significant effect on objective response rate (ORR) (48% versus 23%, P < 0.0001) [21,24]. A blinded independent review of tumour assessments in E2100 confirmed the significant improvement in PFS (median 11.3 versus 5.8 months; HR = 0.48; P < 0.0001) [21] and ORR (50% versus 22%, P < 0.0001) [23]. One-year survival was significantly higher with Bevacizumab (median 81.4% versus 74%; P = 0.017) and there was a trend towards improved OS (26.5 versus 24.8 months; HR = 0.87; P = 0.14) compared with Paclitaxel alone [22].

The combination of Bevacizumab with Docetaxel as first-line therapy for women with LR breast cancer or MBC was evaluated in the phase III AVastin And DOcetaxel (AVADO) trial [23]. A total of 736 patients received Docetaxel (100 mg/m2) in combination with either Bevacizumab (at 7.5 or 15 mg/kg) or placebo every 3 weeks. Treatment with Bevacizumab or placebo was continued until disease progression or unacceptable toxicity; up to nine cycles of Docetaxel were allowed. The primary analysis for AVADO was carried out after a pre-specified number of PFS events and demonstrated significant PFS improvement in both Bevacizumab arms relative to placebo [25]. In the latest analysis of data from this trial, carried out after a median follow-up of 25 months, PFS was superior in both Bevacizumab treatment arms compared with placebo and results for the 15 mg/kg arm were more favourable than for the 7.5 mg/kg arm [median 10.0 months (15 mg/kg), HR = 0.67; P = 0.0002 and 9.0 months (7.5 mg/kg), HR = 0.80; P = 0.0450 versus 8.1 months] [23].

The RIBBON-1 phase III study of 1237 patients has also demonstrated the efficacy and safety of combining Bevacizumab with first-line chemotherapy for LR breast cancer or MBC, including Taxanes [9]. Patients received placebo or Bevacizumab 15 mg/kg every 3 weeks in combination with the physician's choice of Taxanes, an Anthracycline-based regimen or Capecitabine. Irrespective of the chemotherapy partner, the combination with Bevacizumab showed significantly better efficacy than chemotherapy plus placebo [9].

Recently, a meta-analysis of these three randomised trials confirmed that the addition of Bevacizumab to chemotherapy offers a clinically meaningful and statistically significant improvement in PFS (9.2 vs 6.7 months, HR = 0.64 and p = 0.0001) and OR in patients with MBC but does not benefit OS (26.7 vs 26.4 months, HR = 0.97 and p = 0.56) [10].

The side-effect profile of Bevacizumab is well established and generally manageable and is known to differ considerably from those of conventional chemotherapy agents [26]. Adverse events associated with Bevacizumab therapy include hypertension, gastrointestinal perforations, arterial or venous thromboembolic events, cardiotoxicity, fistula/abscess, bleeding events, proteinuria or wound-healing complications. However, the more frequently seen adverse events are mild to moderate in severity, responsive to standard treatment interventions and do not lead to treatment cessation [18].

In phase III studies, the toxicity profiles of Bevacizumab and Taxanes did not overlap and Bevacizumab - Taxane doublets were generally well tolerated [7,23].

The improvement in PFS has not been associated with an OS advantage with the use of Bevacizumab and a Taxane. Although OS is regarded as a "gold standard" of efficacy, the availability of other agents to treat patients with MBC impacts on the difficulty in demonstrating a significant OS benefit from the initial regimen used. Overall, these data indicate that since there is no contraindication or safety issue when combining Bevacizumab with Taxanes, this is a valid treatment option, with advantages over Taxane doublets.

Despite the small number of patients considered in our study, results in terms of PFS are similar to published literature, with an acceptable safety profile.

It should be noted that on 18^th ^November 2011 the US Food and Drug Administration (FDA) released news saying that the agency was revoking the it's approval of the breast cancer indication for Avastin (bevacizumab) after concluding that the drug has not been shown to be safe and effective for that use. It was also indicated that Avastin will still remain on the market as an approved treatment for certain types of colon, lung, kidney and brain cancer (glioblastoma multiforme).

## Conclusion

As demonstrated in this study, Bevacizumab based chemotherapy and particularly the combination of Bevacizumab and Taxanes confirms the benefice on PFS for patients who have not received previously cytotoxic therapy for HER2 negative MBC.

In our opinion, the positive opinion of the Europeen commission to maintain as treatment option the combination Bevacizumab - Paclitaxel in first line treatment of HER2 negative MBC remains therefore valid.

## Competing interests

The authors declare that they have no competing interests.

## Authors' contributions

LB and SB have conceived the study, exploited data, coordinated, drafted, wrote the manuscript, and have performed the statistical analysis. All authors read and approved the final revised version manuscript.

## Financial interests

None.
